# Rapid deracemization through solvent cycling: proof-of-concept using a racemizable conglomerate clopidogrel precursor†

**DOI:** 10.1039/d3cc00332a

**Published:** 2023-02-20

**Authors:** Sjoerd W. van Dongen, Iaroslav Baglai, Michel Leeman, Richard M. Kellogg, Bernard Kaptein, Willem L. Noorduin

**Affiliations:** a AMOLF Science Park 104 Amsterdam 1098 XG The Netherlands bernard.kaptein@innosyn.com noorduin@amolf.nl; b Symeres Kadijk 3 Groningen 9747 AT The Netherlands; c InnoSyn BV Urmonderbaan 22 Geleen 6167 RD The Netherlands; d Van’t Hoff Institute for Molecular Sciences, University of Amsterdam Science Park 904 Amsterdam The Netherlands

## Abstract

We demonstrate that a conglomerate-forming clopidogrel precursor undergoing solution phase racemization can be deracemized through cyclic solvent removal and re-addition. We establish that the combination of slow growth and fast dissolution of crystals is ideal for rapid deracemization, which we achieve by repurposing a Soxhlet apparatus to realize the slow removal and fast re-addition of solvent autonomously.

## Abstract 2

Using a Soxhlet-apparatus, we demonstrate that a conglomerate-forming clopidogrel precursor undergoing solution phase racemization can be deracemized through cyclic solvent removal and re-addition.

## Graphical abstract

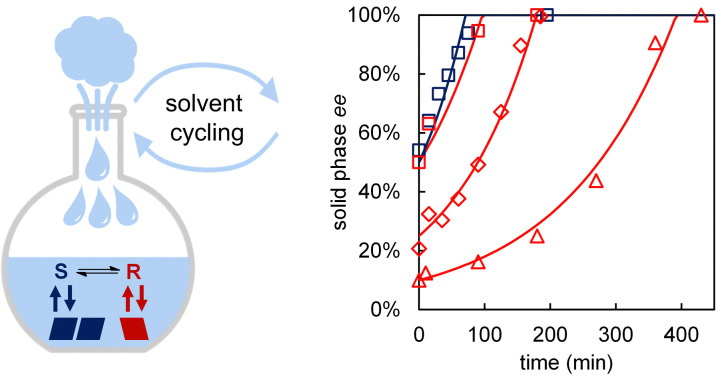

Crystallization-based deracemization processes involving continuous or alternative growth and dissolution of crystals have gained much attention as practical routes for obtaining bioactive enantiopure molecules and as plausible schemes towards prebiotic chiral building blocks.^1–12^ These processes require enantiomers to interconvert *via* a solution phase racemization reaction while both enantiomers crystallize into separate crystals (*i.e.* as racemic conglomerate). When a scalemic mixture of such *R* and *S* crystals undergoes continuous growth and dissolution, the enantiomorphs in minority in the solid phase are converted into the majority phase *via* solution phase racemization. Such asymmetric crystallization processes only stop once full solid-phase enantiopurity has been reached. In the first crystallization-induced deracemizations, continuous growth and dissolution was achieved by grinding slurries of crystals.^2^ Spatial heat gradients and temporal heat fluctuations have subsequently been exploited to grow and dissolve crystals for deracemization.^3–5,10,11,13^ The deracemization process can be sped up by applying more intense grinding, metastable compounds or steeper heating/cooling cycles.^4,6,7,10,11,14,15^ Although grinding is simple and fast, deracemization by temperature cycling may offer the advantage that the growth and dissolution steps are disentangled and can therefore be separately optimized. Specifically, we recently showed that slow growth and deep cooling result in large asymmetric crystallization with virtually exclusive growth of the majority phase.^16^ In contrast, we here realize that fast heating should be favourable to keep the overall cycle time short. Hence, we hypothesize that a process combining slow crystal growth and fast dissolution would be ideal to achieve rapid deracemization. This is challenging, as the quick and homogeneous heating of volumes is often difficult to achieve practically.

Motivated by the above, we propose to grow and dissolve crystals during crystallization-induced deracemization by the removal and re-addition of solvent (Fig. 1). Unlike previously developed deracemization methods, independent control over the rate of solution addition/re-addition in the presented solvent cycling approach should in principle allow for the desirable slow growth and fast dissolution for practical rapid deracemization.

**Fig. 1:**
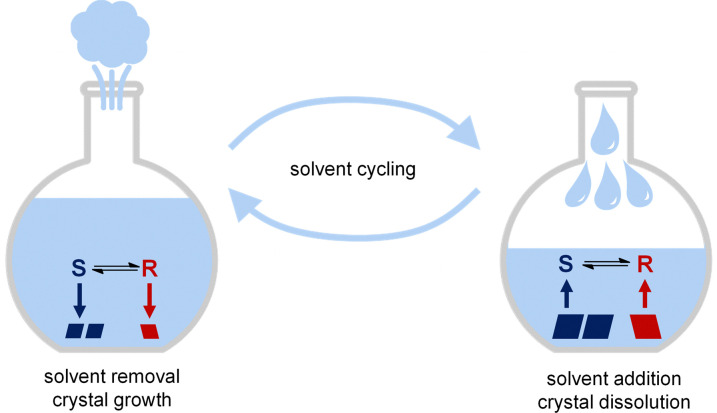
Solvent cycling-induced deracemization relies on alternating removal of solvent (*e.g.* evaporation), during which crystals grow, and re-addition (*e.g.* condensation) of solvent, during which crystals dissolve.

To demonstrate the proof-of-principle of deracemization through solvent cycling, we use the clopidogrel-precursor 2-(benzylideneamino)-2-(2-chlorophenyl)acetamide (1, Fig. 2a). Compound 1 crystallizes as a conglomerate and represents a large class of amino acid Schiff base derivatives that can straightforwardly be racemized in solution using an organic base for crystallization-induced deracemization.^17^ Since 1 has been studied extensively in various crystallization-induced deracemizations, this is an ideal model compound to demonstrate the proof-of-principle and benchmark the performance with previously developed methods.^6,11,16,17^

To realize solvent cycling-induced deracemization experimentally, we repurpose a Soxhlet apparatus (Fig. 2b). Traditionally, a Soxhlet apparatus is used for the continuous extraction of soluble components from solid material placed in the sample compartment.^18^ Here, the Soxhlet apparatus is exploited for the autonomous removal and re-addition of solvent to the slurry. To cycle the solvent, the slurry is gently stirred under reflux, enabling slow solvent evaporation. The evaporated solvent is temporarily collected in the empty sample compartment of the Soxhlet until the maximum level of solvent is collected (<5 min) and the entire volume of evaporated solvent is abruptly re-added (<10 s) to the refluxing slurry. The intrinsic design of a Soxhlet apparatus is thus ideally suited for autonomous solvent cycling with optimal conditions for deracemization: The slow evaporation of solvent results in slow crystal growth which is ideal for large asymmetric crystal growth, while the abrupt addition of solvent causes fast dissolution and therefore shortens deracemization cycles.

**Fig. 2:**
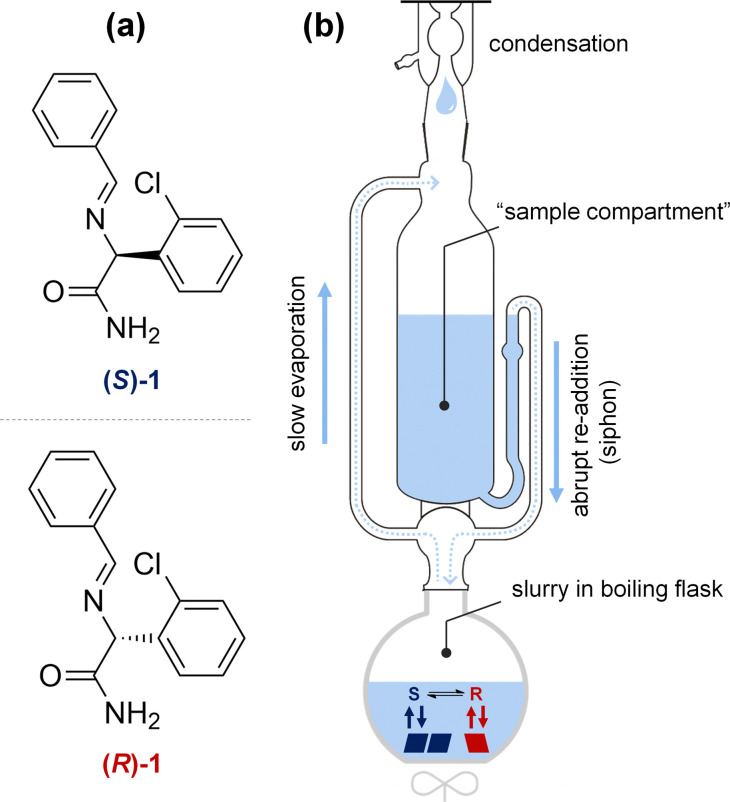
Model compound 1 (a) is deracemized through autonomous solvent cycling by repurposing a Soxhlet apparatus (b). A slurry of *R* and *S* crystals is stirred in a boiling flask containing racemization catalyst. On heating, solvent evaporates and condenses in the sample compartment traditionally used for extraction. When the critical volume is reached, the condensed solvent is abruptly re-added to the boiling flask through a siphoning effect. This results in the desired slow removal and fast re-addition of solvent.

The choice of solvent is crucial to achieve successful deracemization by solvent cycling. Usually, 1 is deracemized in MeCN, but the relatively high boiling point of this solvent (82 °C) has multiple drawbacks. Specifically, at high temperatures, 1 is more prone to undesired side-reactions. Moreover, the solubility is increased to such an extent that large amounts of material are in the liquid phase – rather than the solid phase – thereby lowering yield and efficiency, since liquid phase molecules are not deracemized. Finally, the supersaturation increases rapidly at high temperatures, leading to undesired primary nucleation and fast growth with low chiral enrichment by asymmetric crystal growth.^16^ These considerations highlight the importance of solvent choice for solvent cycling-induced deracemization.

In view of the above, we select a binary solvent mixture of diethyl ether and acetone for the deracemization of 1. Although diethyl ether has the lowest boiling point of all common organic solvents (b.p. = 35 °C), the addition of acetone (b.p. = 56 °C) increases both the solubility and racemization rate. To judiciously balance the solubility and boiling point, we simulate solvent cycling processes with different ratios of solvents based on empirical data that we collect on the boiling points, vapour compositions and solubilities of various binary acetone-diethyl ether solvent mixtures (Fig. 3), (full simulation details provided in the ESI†). Specifically, in these simulations we modulate the ratio between the two solvents to maximize the mass transfer that occurs during each cycle: *i.e.* the amount of solid that is grown during evaporation and subsequently redissolved by re-addition of the solvent, to maximize the deracemization rate.

**Fig. 3:**
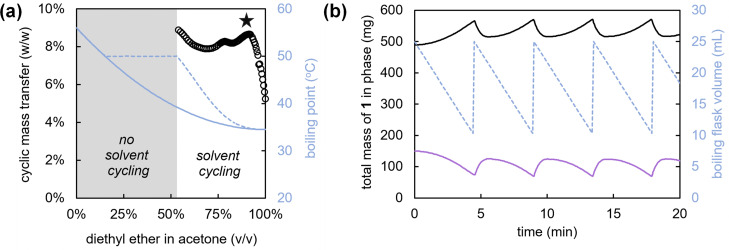
Computer simulation of solvent cycling using a binary solvent mixture at 50 °C. Cyclic mass transfer (black spheres) is computed for a solvent containing various compositions of diethyl ether and acetone (a). The optimal solvent composition (90 vol% diethyl ether) is indicated by a star. The initial and maximum boiling point during the cycle are indicated by solid and dotted blue lines respectively. A sample solvent cycling run with this optimal solvent was simulated and the total mass of 1 in the solid phase (black solid line) and liquid phase (purple solid line) was plotted as function of time. Cycle time is approximately 4.5 minutes. The cycling solvent volume of the boiling flask is indicated with a blue grey line.

For a solvent cycling process operating at 50 °C, we compute the expected mass transfer per cycle as a function of solvent composition (Fig. 3a).‡‡This operating temperature was selected to fit well between the boiling points of the low boiling component (diethyl ether, 35 °C) and high boiling component (acetone, 56 °C). From the simulated runs (Fig. 3b), we project that for low concentrations of acetone in diethyl ether the mixture will lead to insufficient solvation of 1, and therefore less possible total mass transfer between cycles. In contrast, for high concentrations of acetone, we find that the boiling point increases so much that an insufficient amount of solvent evaporates. A mixture of 90 vol% diethyl ether and 10 vol% acetone provides the desired balance between a low boiling point and a large mass transfer per cycle (Fig. 3a).

Guided by the simulations, we experimentally demonstrate solvent cycling-induced deracemization. We first suspend 800 mg of 1 with 10% ee in (*R*)-1 in 10 mL of the designed binary solvent mixture (90 vol% diethyl ether and 10 vol% acetone). This suspension is sonicated for 30 minutes to obtain a homogenous slurry. The slurry is then transferred to a 50 mL round-bottom flask, containing 15 mL more solvent as well as PTFE spheres to avoid uneven boiling and caking. The flask is immersed in a water bath, gentle stirring is applied, and the flask is fitted with a Soxhlet apparatus with condenser (15 mL sample compartment volume), allowing for cyclic condensation and immediate re-addition of the evaporated solvent every 4 to 5 minutes. After heating the water bath to 50 °C and addition of the racemization catalyst (40 μL of 1,8-diazabicyclo[5.4.0]undec-7-ene (DBU)), we already observe virtually complete deracemization of the solid within 18 hours, from initially 10% to 99% ee in (*R*)-1 with 90% yield.

We determine the deracemization kinetics by performing solvent cycling experiments starting with 10%, 20% and 50% initial ee in (*R*)-1 (Fig. 4). These kinetics show that full deracemization can be achieved within 2, 3.5, and 7 hours respectively. The enantiomeric enrichment of the solid phase follows sigmoidal amplification of the ee, which is typical for this type of crystallization-induced enantiomeric transformations. As expected, starting with 50% ee in (*S*)-1 also leads to complete deracemization in (*S*)-1 with matching deracemization kinetics, indicating that the process has no substantial (kinetic) bias towards any of the two enantiomers (Fig. 4).

**Fig. 4:**
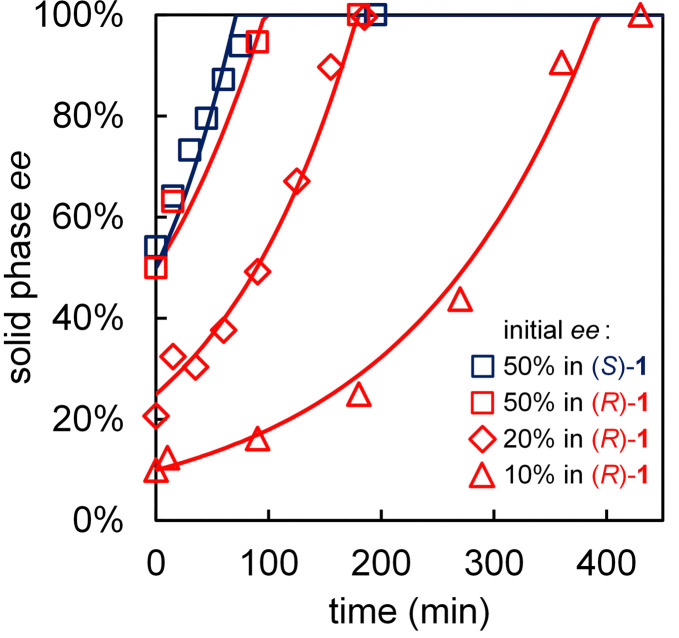
Deracemization kinetics of 1 by solvent cycling-induced deracemization (800 mg in 25 mL solvent (9 : 1 diethyl ether/acetone) containing 40 μL DBU at 50 °C) for various initial ee. Exponential kinetic fits are represented by solid lines.

To validate that the fast deracemization kinetics are not caused by metastable crystal transformations or attrition, we perform several control experiments.^2,15^ First, comparing powder crystal patterns of the equilibrated slurry and the final product shows identical patterns, indicating that the presence of metastable polymorphs or solvates as key intermediates in the deracemization can be excluded (diffractograms provided in the ESI†). Second, comparing the kinetics of an experiment with and without Soxhlet apparatus (but with condenser and PTFE beads), shows that virtually no deracemization is observed over the relevant timelines without the Soxhlet apparatus. This suggests that neither attrition by stirring, nor the presence of the PTFE beads, but solvent cycling instead induces the observed deracemization (data shown in the ESI†).

We benchmark the performance of the here-introduced solvent cycling process against well-established deracemization by attrition-enhanced deracemization and temperature cycling. Typically, using attrition-enhanced deracemization and temperature cycling, starting material of 20% ee in 1 deracemizes in approximately 10 hours.§§Attrition-enhanced deracemization has been achieved within 45 minutes using a bead mill setup, but such equipment is oftentimes not readily accessible, and not all compounds can withstand the associated vigorous grinding conditions.^6^ ^6,11,17^ In contrast, using solvent cycling, starting material of 20% ee completely deracemizes in only 3.5 hours (Fig. 4). Hence, even without any extensive optimization, solvent cycling-induced deracemization can readily outperform well-established deracemization processes.

In summary, we introduce solvent cycling as a robust and rapid deracemization method. Based on the key insight that slow and long crystal growth, and fast crystal dissolution are desirable for efficient deracemization, we implement solvent cycling autonomously and efficiently through repurposing a Soxhlet apparatus. To demonstrate the proof-of-principle, we develop a simulation-guided solvent design strategy to tailor a binary solvent mixture that maximizes mass transfer while maintaining a low boiling point during the deracemization. By combining these insights, we show that solvent cycling-induced deracemization—even in nonoptimized form—is simple and more efficient in execution than most reported crystallization-induced deracemization techniques.

Our results emphasize the importance and potential of individually controlling the rates of crystal growth and dissolution for crystallization-induced deracemizations. We foresee new and synergistic opportunities for our solvent-tailoring strategy to accurately control and optimize key deracemization parameters (*e.g.* crystal growth and dissolution rates, amount of cyclic mass transfer, racemization rate), allowing the design of new deracemization systems that were previously ineffective or impossible. Preferentially cycling a ‘good’ solvent would be especially interesting to explore, exploiting an inverse anti-solvent effect.

The versatility and tunability of solvent cycling readily allow for extending these principles to other chiral compounds by tailoring the solvent, racemization catalyst or—if necessary—inserting a racemization loop.^6,8,9,12^ Moreover, optimization can for instance be achieved by implementing flow control technologies or similar physical mechanisms for fine-tuning the regulation of solvent removal and re-addition rates. Besides optimization, many variants on the practical realizations of the removal and re-addition of solvent may be realized. In fact, during the preparation of this work, Flood *et al.* developed an alternative form of solvent cycling using evaporation by reduced pressure.¶¶Both the study of Flood *et al.* and this work were performed independently and both studies were completed without each other's knowledge. Only during the preparation did the authors of both manuscripts become aware of the related work.^19^ ^19^ In addition, we foresee that solvent cycling processes can readily be scaled-up in conventional equipment, adapted in a continuous fashion, and implemented for the practical production of enantiopure molecules.^13,20,21^

Many types of stimuli can be exploited for crystallization-induced deracemizations; oscillating electrochemical potentials, alternating pressures or pH, photo-switchable reactions, and electromagnetic oscillations are just some of the many ways to induce continuous or alternating crystal growth and dissolution. Also, our results show the importance of cyclic physical chemical processes in synthesizing chiral molecules for practical purposes as well as in origin of life scenarios.

SWvD and WLN acknowledge OCENW.KLEIN.155, which is financed by the Dutch Research Council (NWO).

## Conflicts of interest

There are no conflicts to declare.

## Supplementary Material

CC-059-D3CC00332A-s001
