# Supplementary material for: Rapid deracemization through solvent cycling: proof-of-concept using a racemizable conglomerate clopidogrel precursor
Source: Chem Commun (Camb). 2023 Feb 20;59(26):3838–41. doi: 10.1039/d3cc00332a (PMC10043878; doi:10.1039/d3cc00332a)
Supplement: CC-059-D3CC00332A-s001 [file CC-059-D3CC00332A-s001.pdf]

Electronic Supplementary Material (ESI) for ChemComm.
This journal is © The Royal Society of Chemistry 2023

Supporting Information

Rapid Deracemization through Solvent Cycling: Proof-of-Concept using a Racemizable Conglomerate Clopidogrel Precursor

Sjoerd W. van Dongen[a], Iaroslav Baglai[a], Michel Leeman[b], Richard M. Kellogg[b], and Bernard Kaptein*[c], Willem L. Noorduin[a,d]*

[a] AMOLF, Science Park 104, 1098 XG, Amsterdam, The Netherlands.

[b] Symeres, Kadijk 3, 9747 AT, Groningen, The Netherlands.

[c] InnoSyn BV, Urmonderbaan 22, 6167 RD, Geleen, The Netherlands.

[d] Van 't Hoff Institute for Molecular Sciences, University of Amsterdam, Science Park 904, 1098 XH, Amsterdam, The Netherlands.

* Email: bernard.kaptein@innosyn.com; noorduin@amolf.nl

Table of Contents

	Experimental Details	p. S-2
A.	General remarks	p. S-2
B.	HPLC analysis	p. S-2
C.	Solvent Cycling-Induced Deracemization using a Soxhlet-Apparatus	p. S-3
D.	X-Ray Powder Diffraction Analyses	p. S-6
E.	Simulations for the Design of the Binary Solvent Mixture	p. S-7
	References	p. S-10

Experimental Details

A. General remarks

Chiral HPLC analyses were performed using an Agilent Technologies Infinity 1260 HPLC system. All solvents used in HPLC analysis (n-heptane, 1-propanol, acetonitrile) were HPLC grade (≥ 99%) and obtained from VWR chemicals. Solvent for the binary solvent mixture (acetone, diethyl ether) was AR-grade (biosolve, VWR). For sample preparation and analysis, 0.2 µm PTFE syringe filter (VWR Internationals) and 2 mL HPLC vials (29651-U Supelco, Merck) were used. When stirring, cylindrical PTFE stirring bars (15x6mm, oval rare earth, VWR) combined with a standard VWR hotplate were used. The PTFE beads (3 & 6 mm PTFE balls, BOLA) and glass beads (2 mm glasschrot, Assistent) were ordered through VWR. The racemization catalyst used is 1,8-Diazabicyclo[5.4.0]undec-7-ene (Across Organics). The used Soxhlet-apparatus has a nominal chamber size of 30 mL, which was decreased to 15 mL by inserting a 22 mL vial inside the sample compartment (vial: 22 mL (27172-U Supelco); Soxhlet: 30 mL (537-0047 VWR)). Racemic and enantiopure starting material of compound **1** was obtained over the course of previous research [1].

B. HPLC analysis

<u>Sampling and sample preparation</u>

Two distinct sampling methods were used. One method for system sampling and one method for solid phase sampling. For both methods, samples are always taken at the end of each solvent cycle (i.e. directly after re-addition, when the solvent in the boiling flask is at maximum).

- System sampling

A 1 mL syringe fitted with a long needle is used to collect ~ 50 µL of slurry from the boiling flask. This system sample is then dissolved in 7 mL of IPA. After brief ultrasonication (5 minutes), 1 mL is transferred to a 2 mL HPLC vial and directly submitted for HPLC analysis.

- Solid phase sampling

A 1 mL syringe fitted with a long needle is used to collect ~ 250 µL of slurry from the boiling flask. The collected suspension was cast on top of filter paper laid down on glass filter connected to a vacuum filtration set-up (whilst under active vacuum). Two separate samples of the solids were taken using a Pasteur pipette, dissolved in 1.5 mL of IPA by vortex and ultrasonication (10 minutes, 2 mL HPLC vial) and subsequently analysed by HPLC.

<u>HPLC method</u>

HPLC analysis was performed on a chiral column (CHIRALPAK IA (250 x 4.6 mm, 5µm)) with a mobile phase consisting of n-heptane and 1-propanol. For compound **1** (Fig. S-1), the eluent is mixed in a 7:3 ratio (heptane:IPA). The flow rate was 0.7 mL/min, injection volume 4 µL, and detection was performed by UV-detector (wavelength: 220 nm). Each run had a total time of 12 minutes.

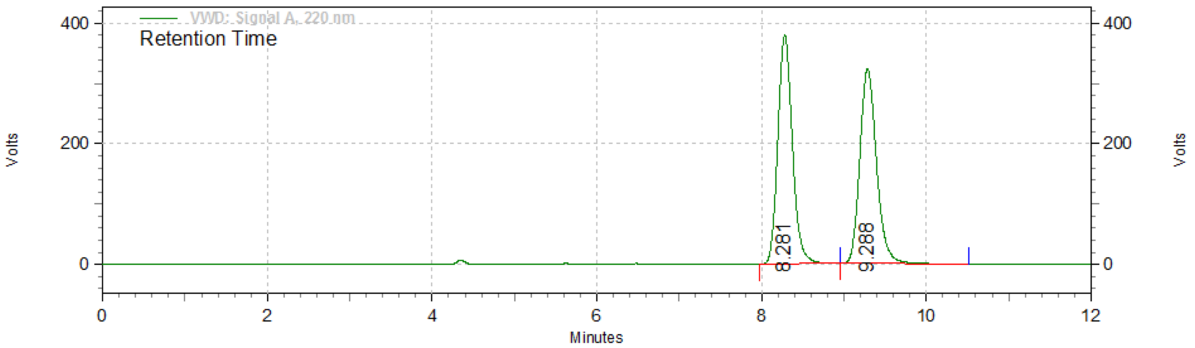

Fig. S-1. Typical chromatogram with retention times: 8.28 min for (R)-**1**, and 9.29 min for (S)-**1**.

C. Solvent Cycling-Induced Deracemization using a Soxhlet-Apparatus

Preliminaries

For the solvent cycling experiments, we use a two-neck round bottom flask of 50 mL, to which we attach a Soxhlet-apparatus and a tap-water cooled condenser. The other neck is closed with a septum to allow sampling during the solvent cycling process. The Soxhlet-apparatus has a nominal volume of 30 mL, which is decreased to 15 mL by adding a closed glass vial to the sample compartment. Aluminium foil is used to prevent unwanted cooling of the glassware by air during the experiments. A batch of 250 mL of the binary solvent mixture (225 mL diethyl ether and 25 mL acetone) was prepared for use throughout the various experiments. During the solvent cycling experiments, unless stated otherwise, 28 g of PTFE beads and a stirring bar are present in the round-bottom flask (boiling flask) and stirring is performed on the highest possible setting. Experiments are performed under slight nitrogen pressure (Schlenk line) to reduce the possibility of solvent evaporation out of the system as far as possible.

Slurry preparation

Starting material of **1** of a certain enantiomeric composition was prepared by mixing the required amounts of enantiopure **1** and racemic **1** in a pestle and mortar and ground until homogenous. The resulting *ee* of the starting material was always confirmed by HPLC (an example is shown in Fig. S-2).

To prepare the slurry, a 20 mL vial is charged with 800 mg of compound **1**, in the desired enantiomeric composition. Subsequently, 10 mL of the solvent is added and the resulting suspension is sonicated for 30 minutes to obtain a homogenous slurry. The slurry is then transferred to the 50 mL round-bottom flask and an extra 15 mL of solvent is added to the flask.

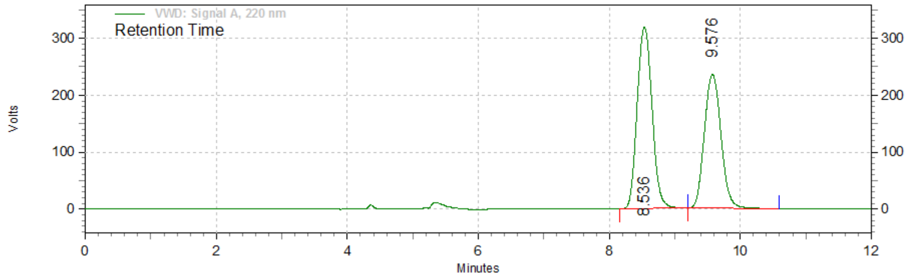

VWD: Signal A, 220 nm Results

Retention Time	Area	Area %	Height	Height %
8.536	84766089	54.88	5338022	57.53
9.576	69683447	45.12	3940171	42.47
Totals	154449536	100.00	9278193	100.00

Fig. S-2. Typical solid phase chromatogram of the initial seed material (ee_0 = 10%).

Seminal solvent cycling experiment

Starting material of 10% *ee* in (R)-**1** was used to prepare the slurry. The round-bottom flask was connected to the Soxhlet-apparatus and the condenser as described and lowered into a water bath set at 50 ^{o}C on a hotplate (maximum stirring setting). Once the solvent cycles attained a consistent time-interval (approximately 4 to 5 minutes per cycle), 40 µL of racemization catalyst (DBU) was added in 2.5 mL of solvent by syringe through the septum. After 18 hours, the solid phase and system were sampled as previously described. The seminal solvent cycling experiment was deemed successful, since virtually all material had converted to the major enantiomer. The final solid phase chromatogram is displayed as Fig. S-3 (>99% ee in (R)-**1**). After cooling back to 20 ^{o}C, the contents of the boiling flask were filtered to afford virtually enantiopure material with 90% yield as a white residue.

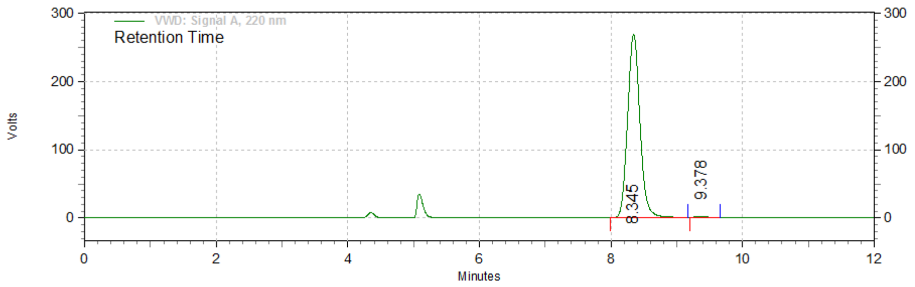

VWD: Signal A, 220 nm Results

Retention Time	Area	Area %	Height	Height %
8.345	58620914	99.56	4504637	99.55
9.378	260804	0.44	20425	0.45
Totals	58881718	100.00	4525062	100.00

Fig. S-3. Typical final solid phase chromatogram following a successful solvent cycling-induced deracemization experiment (t = 18 hrs, ee_0 = 10%).

Kinetics of Solvent Cycling-Induced Deracemization

Having demonstrated the potential of Solvent Cycling-Induced Deracemization, the experiment was repeated with starting material of 10%, 20% and 50% ee in (R)-**1** and 50% in (S)-**1**. At various points after starting the experiment, solid phase and system samples were taken (Fig. S-4). These experiments show that full deracemization can be achieved within 2, 3.5, and 6 hours respectively.

N.B. The system phase *ee* is always lower than the solid phase *ee* since a number of molecules is in the racemic liquid phases (soluble). This is also a reason why solubility should not be too high while cyclic mass transfer should be optimized.

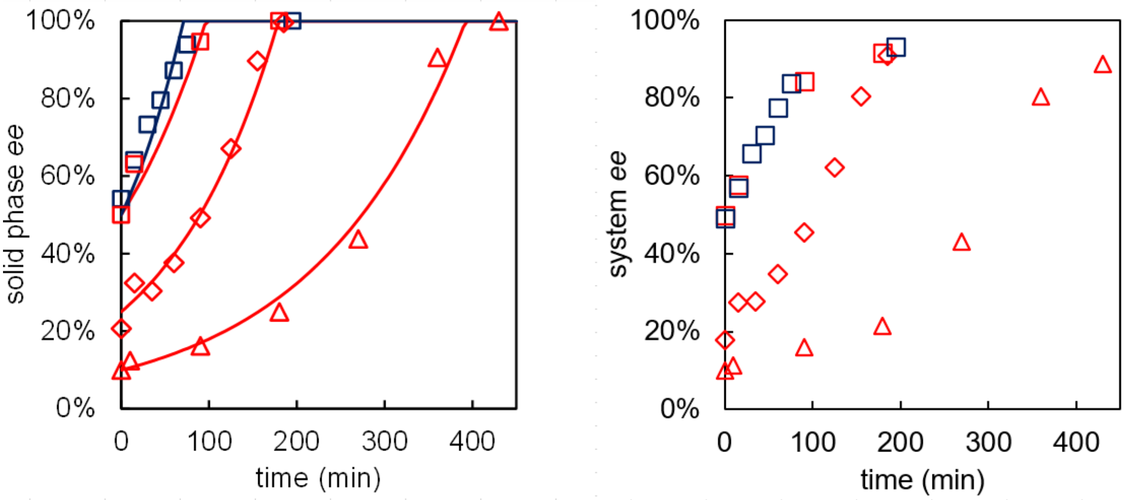

Fig. S-4. Deracemization kinetics of **1** by solvent cycling-induced deracemization. Initial *ee*: 10% (triangles), 20% (diamonds) and 50% (squares) in R (red) or S (blue). Exponential kinetic fits are represented by solid lines. The left graph shows the solid phase *ee* and the right graph shows the system *ee*.

Control experiments
In order to confirm that solvent cycling is the cause of the enantiomeric enrichment observed, and not–for example–attrition by the PTFE beads or stirring or ripening phenomena accelerated at higher temperatures, we conducted two specific control experiments. The kinetic solvent cycling experiment was repeated starting with material 10% *ee* in (R)-**1**.

In both experiments, the Soxhlet-apparatus is removed and the condenser is placed directly on top of the boiling flask. In this way, no–or only minimal trough refluxing–solvent cycling is achieved. These experiments were then performed in the presence of either the PTFE spheres or glass beads, since glass beads were used in the first demonstration of attrition-enhanced deracemization [2].

The results are shown in Fig. S-5. Comparing of these experiments clearly demonstrates that solvent cycling, using the Soxhlet-apparatus, is vastly superior in kinetics to the two control experiments. In fact, on the relevant timescales of deracemization through solvent cycling, almost no enrichment is produced by the control experiment with PTFE beads and refluxing alone. This shows that it is the removal and re-addition of the solvent that is responsible for the enantiomeric enrichment reported here. Moreover, attrition by glass beads clearly outperforms the PTFE spheres (but still does not reach the kinetics of solvent cycling by any stretch). This shows that the soft PTFE beads are no strong source of attrition as compared to glass beads, the standard in attrition-enhanced deracemization experiments, and their impact is indeed minimal.

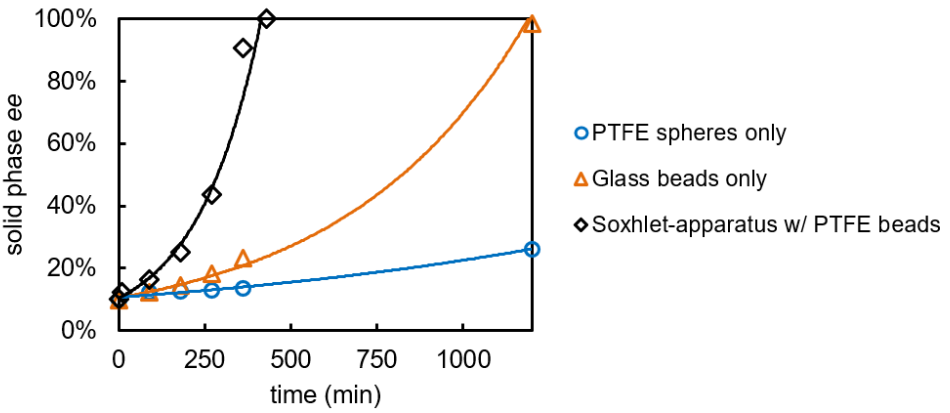

Fig. S-5. The deracemization kinetics were determined (starting with 10% *ee* in the solid phase) for three experiments: solvent cycling with a Soxhlet apparatus containing PTFE beads in the boiling flask (black diamonds), refluxing (no actual solvent cycling) with glass beads only (orange triangles), and refluxing with PTFE spheres only (blue circles). The solid lines are exponential kinetic fits.

D. X-Ray Powder Diffraction Analyses

In order to exclude the presence of metastable polymorphs or solvates as key intermediates in the deracemization and confirm that no new polymorphs were formed as a starting material or final product during solvent cycling in the designed solvent, X-Ray Powder Diffraction analyses were performed on racemic reference material of **1** (unexposed), the sonicated initial slurry (before starting the solvent cycling experiment, but having been exposed to the solvent) and the final deracemized product (having been exposed to the process). For each sample, 20 – 50 mg of powder was placed on a 1 cm^2 aluminium square and pressed into a cake. X-Ray powder diffraction patterns of each sample were measured using a Bruker D2 Phaser with a Cu X-ray source (Cu K-α, λ = 1.5418 Å). The resulting patterns are displayed in Fig. S-6. These patterns are identical for all three samples, indicating that the presence of metastable polymorphs or solvates as key intermediates in the deracemization can be excluded and no new polymorphs were formed as a starting material or final product during solvent cycling in the designed solvent.

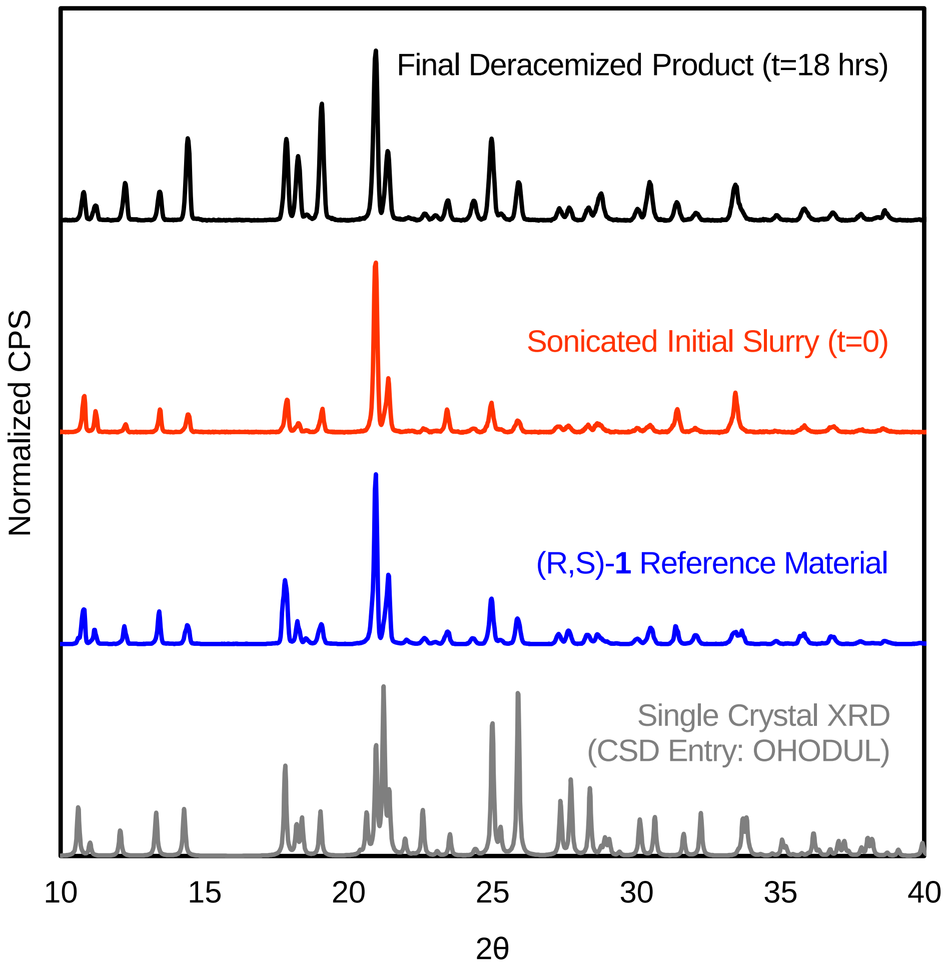

Fig. S-6. XRPD patterns for the three samples of interest: reference material of (R,S)-**1**, the sonicated initial slurry (before starting the solvent cycling experiment, but having been exposed to the solvent) and the final deracemized product (having been exposed to the process). We have also included a Single Crystal XRD (simulated XRPD) reference from the existing CSD entry OHODUL [3].

E. Simulations for the Design of the Binary Solvent Mixture

In order to design a solvent with tailor-made composition which has a low boiling point and relatively low solubility at the boiling point, while still providing reasonably fast racemization kinetics, we use a binary solvent mixture of diethyl ether and acetone. To guide our choice in composition, we have simulated solvent cycles for various compositions, boiling points and solvent cycling geometries.

Our model consists of two compartments: the boiling flask, from which solvent is removed by evaporation, and the sample compartment, which adds all solvent condensed back to the boiling flask once a critical volume is reached – analogous to the Soxhlet-apparatus set-up.

Premise and Assumptions

In our model, we take a range of considerations into account. Not only the properties of the bare solvent are relevant. Since the solvent composition changes during the process, also the properties during the deracemization process should be considered. This means that:

- ✓ We consider that the composition of the vapour (the evaporated solvent) depends on the composition of the solvent that is boiling (in the boiling flask).
- ✓ We consider that the composition in the boiling flask changes as a result.
- ✓ We consider that the actual solubility of compound **1** and the boiling point of the solvent in the boiling flask changes over time as the composition of the solvent changes.
- ✓ We consider that that changes in solubility and volume mean that crystals have to grow or dissolve, but these processes are not instantaneous and have a mass transfer rate.

The relative amount of cyclic mass transfer is small compared to the total concentration in the solvent. We assume that changes in the boiling point and vapour pressure as a function of solute concentration can thus be neglected for these simulations.

Underlying Datasets

In order to accurately simulate the solvent cycling experiment for the binary diethyl ether – acetone system, we required solubility data as a function of solvent composition. Therefore, we determined the solubility for various compositions and temperatures of the solvent using the method previously reported [1]. The data, shown in Fig. S-7, were taken as empirical basis for the simulations.

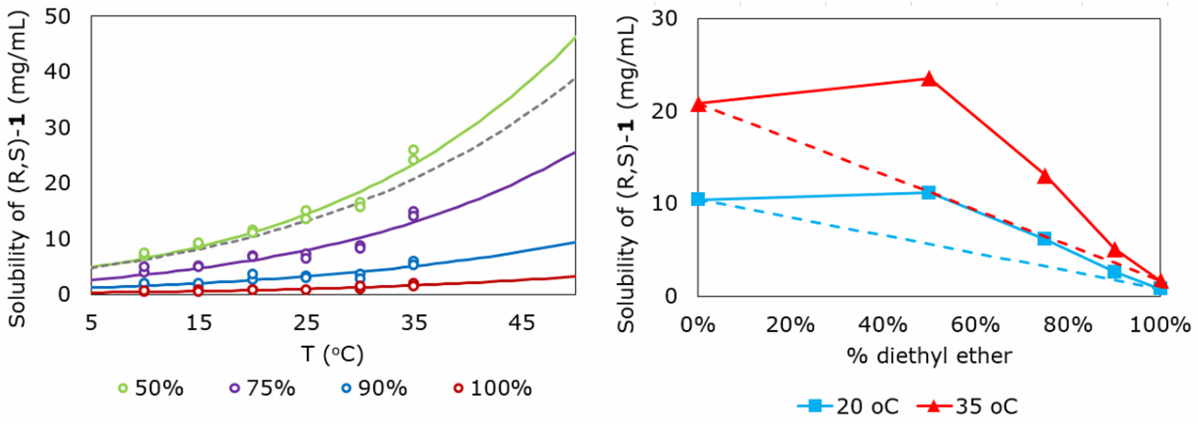

Fig. S-7. The solubility of (R,S)-**1** was determined as a function of temperature (10 – 35 degrees Celsius) and composition (0, 50, 75, 90, 100% diethyl ether in acetone). Data was fitted based on the Van 't Hoff equation (solid lines). The relationship as a function of composition is nonlinear, as visualized on the right: a certain degree of agonism is present. The grey dotted line indicates pure acetone.

Apart from solubility, we also required knowledge on the composition of the vapour of solvent that is boiling and the actual boiling point as a function of solvent composition. This data is modelled based on literature values and visualized in Fig. S-8 [4].

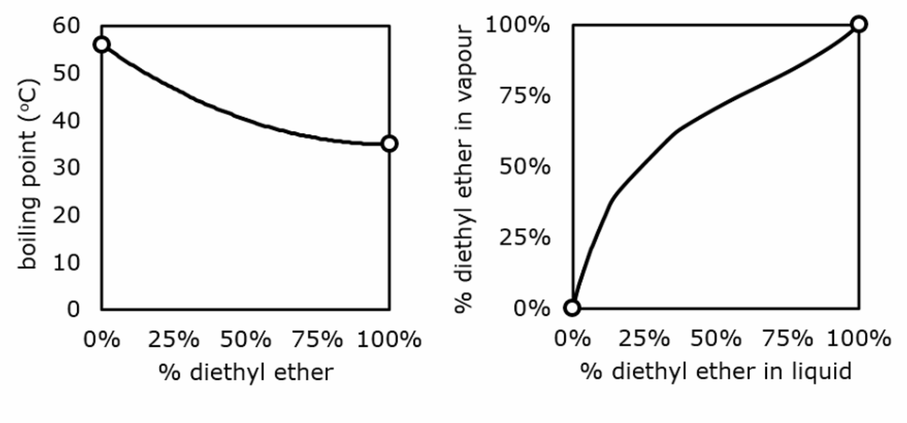

Fig. S-8. The boiling point (left) and vapour-liquid equilibrium (VLE) (right) of the diethyl ether – acetone system are shown, calculated based on literature values [4].

<u>Equations and Implementation</u>

The simulation was implemented in R by following a time-marching approach [R Core Team (2021). R: A language and environment for statistical computing. R Foundation for Statistical Computing, Vienna, Austria. URL https://www.R-project.org/.]. To this aim, the following equations and methods were used to calculate the various variables at each time-step.

- Liquid-Vapour Equilibrium

Composition of the vapour (solvent removed *via* evaporation) was calculated from the composition of the liquid (solvent in the boiling flask) based on an interpolation of the VLE data shown in Fig. S-8. Interpolation was performed using the `splinefun` method in R using a `monoH.FC` method.

- Boiling point

To calculate the boiling point of the solvent in the boiling flask, we use the approximation:

$$\text{boiling point} = (H - L) \cdot \varphi^2 - 2 \cdot (H - L) \cdot \varphi + L$$

where H is the boiling point of the high boiling solvent (acetone), L is the boiling point of the low boiling solvent (diethyl ether) and φ is the binary solvent composition (between 0 and 1).

- Solubility

First, composition dependent ΔH and ΔS were extracted from the solubility data shown in Fig. S-7. Second, the ΔH and ΔS for the actual composition of the solvent in the boiling flask was calculated by interpolation between the known values (similar method as above for VLE). Then, the solubility was predicted based on the calculated ΔH and ΔS for the actual composition. The relationship between solubility and the thermodynamic parameters ΔH and ΔS is given by the Van 't Hoff relationship:

$$\text{solubility} = \rho \cdot \exp\left((-\Delta H/(R \cdot T)) + (\Delta S/R) \right)$$

where ρ is the density of the solvent (function of composition), R is the gas constant and T is the temperature in K.

- Removal and re-addition of solvent

Material is removed from or re-added to the boiling flask using the following mass transfer equation:

$$dV/dt = \text{rate of mass transfer}$$

where the rate is either that of removal or re-addition. The addition or removal in case of the sample compartment follows the negative sign to uphold the mass balance.

- Dissolution and Growth of crystals

For crystal dissolution and growth, the supersaturation is first calculated based on solubility:

$$S = [(R, S)\text{-}\mathbf{1}] / \text{solubility}$$

If $S < 1$, dissolution will take place. If $S > 1$, growth will take place. These processes are modelled following the approximation

$$dm/dt = \text{rate}_{growth} \cdot (S - 1)/(S_{max} - 1) \quad [\text{for growth, if } S < S_{max}]$$

$$dm/dt = \text{rate}_{growth} \quad [\text{for growth, if } S > S_{max}]$$

$$dm/dt = \text{rate}_{dissolution} \cdot S \quad [\text{for dissolution}]$$

where S_{max} is the so-called ‘maximum supersaturation’ at which the steady-state growth rate is reached and dm/dt is the mass transfer rate between solid and liquid phase.

- Time-marching implementation

Separate volumes are tracked for acetone and diethyl ether for each of the two compartments in the implementation. At time-step 0, all parameters in the system are initialized and initial compositions are set. For each new time-step, the procedure is as follows.

First, we calculate the boiling point. If the boiling point is below or equal to the system temperature, we execute a solvent removal step. The amount of solvent to be removed is calculated and the composition of the removed solvent is based on the VLE. The removed solvent is then added to the sample compartment.

Second, we check whether the volume in the sample compartment is above the critical volume or if re-addition was busy in the previous step (if the volume of the sample compartment is at or below the ‘zero-volume’, we stop re-addition). If re-addition is required, we move part of the solvent from the sample compartment back into the boiling flask.

Third, we recalculate compositions of the boiling flask and the sample compartment. Based on this new data, we calculate the solubilities and execute crystal growth or dissolution.

- Simulation parameters

Simulations were nominally performed with the following parameters. Temperature of the system was set at 50 ^{o}C. We used a sample compartment of 15 mL, initial solvent volume in the boiling flask of 25 mL and 1 mL of zero-volume (vapour lost in the system). We routinely used a maximum growth rate of 0.1 mg/s, dissolution rate of 1 mg/s, maximum supersaturation of 1.5 (c/c*), 1 mL/s solvent re-addition rate and 0.01 mL/s solvent removal rate. The amount of compound in the simulations was set at 800 mg. For time-marching, we used 15000 timesteps and each timestep was 0.5 seconds.

References

[1] van Dongen, Sjoerd W., et al. "Chiral Amplification through the Interplay of Racemizing Conditions and Asymmetric Crystal Growth." *Journal of the American Chemical Society* 145.1 (2023): 436.

[2] Noorduin, Wim L., et al. "Complete Deracemization by Attrition-Enhanced Ostwald Ripening Elucidated." *Angewandte Chemie* 120.34 (2008): 6545-6547.

[3] Van der Meijden, Maarten W., et al., CCDC 734087: Experimental Crystal Structure Determination, 2010, DOI: 10.5517/ccsmw7h.

[4] Sameshima, Jitsusaburo. "On The System Acetone—Ethyl Ether." *Journal of the American Chemical Society* 40.10 (1918): 1482-1503.
